# Differential immune signatures in the tumor microenvironment are associated with colon cancer racial disparities

**DOI:** 10.1002/cam4.3753

**Published:** 2021-02-09

**Authors:** Thomas Curran, Zequn Sun, Brielle Gerry, Victoria J. Findlay, Kristin Wallace, Zihai Li, Chrystal Paulos, Marvella Ford, Mark P. Rubinstein, Dongjun Chung, E. Ramsay Camp

**Affiliations:** ^1^ Department of Surgery Medical University of South Carolina Charleston SC USA; ^2^ Ralph H. Johnson VA Medical Center Charleston SC USA; ^3^ Department of Public Health Sciences Medical University of South Carolina Charleston SC USA; ^4^ Department of Pathology and Laboratory Medicine Medical University of South Carolina Charleston SC USA; ^5^ Department of Microbiology and Immunology Medical University of South Carolina Charleston SC USA; ^6^ The Pelotonia Institute for Immuno‐Oncology Ohio State University Comprehensive Cancer Center – James Columbus OH USA; ^7^ Department of Surgery/Department of Microbiology and Immunology The Winship Cancer Institute of Emory University Atlanta GA USA; ^8^ Department of Biomedical Informatics The Ohio State University Columbus OH USA; ^9^ Department of Surgery Baylor College of Medicine Houston TX USA; ^10^ Michael E. DeBakey VA Medical Center Houston TX USA

**Keywords:** colon cancer, immunogenomics, immunology, immunophenotype, racial disparities

## Abstract

**Background:**

Disparities in colon cancer (CC) outcomes may be due to a more aggressive phenotype in African American patients in the setting of a decreased tumor immunity, though the precise mechanism for this result has not been well elucidated. To explore the molecular factors underlying CC disparities, we compared the immunogenomic signatures of CC from African American and European American patients.

**Methods:**

We identified all CC patients from the publicly available Cancer Genome Atlas for whom race and survival data are available. Immunophenotype signatures were established for African American and European American patients. Comparisons were made regarding survival and a multivariable linear regression model was created to determine the association of immune cellular components with race. Differential gene expression was also assessed.

**Results:**

Of the 254 patients identified, 58 (23%) were African American and 196 (77%) were European American. African American patients had a decreased progression free survival (*p* = 0.04). Tumors from African American patients displayed a reduced fraction of macrophages and CD8^+^ T cells and an increased fraction of B cells compared with tumors from European Americans. Differences persisted when controlling for sex, age, and disease stage. Immunostimulatory and immunoinhibitory gene profiles including major histocompatibility complex expression differed by race.

**Conclusions:**

Differences in the tumor immune microenvironment of African American as compared to European American CC specimens may play a role in the survival differences between the groups. These differences may provide targeted therapeutic opportunities.

## Graphical abstract

We uncovered key molecular factors underlying racial disparities in African American versus European American patients. Immunogenomic signatures in these patient cohorts were identified using a publicly available tumor repository and robust bioinformatic tools. Significant differences in gene expression were found in the tumor microenvironment which may underlie key clinical differences in these patients and reveal new therapeutic targets to effective treat all patients in the future.

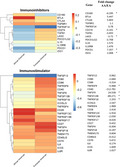

## 1 BACKGROUND

Colorectal cancer (CRC) is the third most common malignancy, and the second leading cause of cancer deaths in the United States.^1^ Colon cancer (CC) specifically accounts for over 70% of new CRC cases in the United States annually.^1^ Compared to European Americans (EAs), Blacks with African ancestry (AAs) have a substantially higher (30%–50%) CRC mortality rate, marked by both higher incidence and lower survival rate.^2^ Socioeconomic factors and absence of preventive medical care likely contribute to the heightened incidence, reduced early detection, and delayed treatment. However, CRC racial health disparity remains significant despite improved CRC screening in AA patients.^3^, ^4^, ^5^, ^6^, ^7^, ^8^

Growing evidence has demonstrated that both colon and rectal cancers in AAs have unfavorable tumor biology. A 2018 study by Sineshaw and colleagues^9^ using the National Cancer Database including both colon and rectal cancer found that while access to care and tumor stage accounted for three quarters of the AA‐EA survival disparity in patients under 65, fully 25% of the survival difference remains unexplained suggesting a role for tumor biology underlying the differences in survival. For instance, variations exist in AA patients, specifically in mutations of the mismatch repair genes, PIK3CA and the *p53* tumor suppressor gene which may manifest in disease behavior.^10^, ^11^ Recent evidence in breast and prostate cancer has implicated underlying racial differences in inflammation and immunity as key drivers of the respective cancer disparities.^12^, ^13^ Two studies investigating CC have also highlighted a role for tumoral immunity underlying cancer racial disparities. One study that performed gene expression profiling of CC patients determined that prominent differences were observed in pathways related to inflammatory and cell‐mediated immune response between AAs and EAs.^11^ In addition, a second study of both colon and rectal cancer demonstrated decreased antitumoral cytotoxic immunity suggested by reduced Granzyme B+T cell population among AA patients.^4^ Thus, AA CC, as well as the closely related rectal cancer, patients have altered immunity, although the underlying mechanism has not been determined.

The Cancer Genome Atlas (TCGA) and large CRC molecular profiling analyses have defined distinct CRC molecular subtypes related to anatomy and tumoral immunity.^14^, ^15^, ^16^, ^17^ In parallel, landmark studies have established the prognostic importance of the quantity and quality of the CRC tumor infiltrating lymphocyte.^18^, ^19^ Novel secondary analyses have used TCGA to identify specific immunogenomic gene signatures to describe the tumor immune microenvironment based on unique gene signatures identifying specific cell types.^20^, ^21^ CRC immunogenomic subtypes have effectively correlated with long‐term survival as well as predicted response to immune checkpoint inhibitors.^20^ To explore molecular aspects underlying CRC cancer disparities, we compared the immunogenomic signatures of CC from AA and EA patients.

## 2 METHODS

### 2.1 Patients/Data sources

The Cancer Genome Atlas data for CC previously analyzed by Thorsson and colleagues were made publicly available through the National Cancer Institute Genomic Data Commons.^21^ These data were then integrated with genomic and clinical data available in the cBioPortal for Cancer Genomics, an online platform designed to facilitate access to complex cancer genomics data.^22^ We identified all CC patients from TCGA with immunophenotype data for whom race and survival data were available. Patients with unavailable immunophenotype, race, or survival data were excluded.

### 2.2 Characterization of immunophenotype – TCGA

The leukocyte composition associated with each CC sample within TCGA was previously characterized as an immune cellular fraction using CIBERSORT, a method of estimating proportions of cell types from gene expression profiles.^21^, ^23^ Cell types were considered as an immune cellular fraction and compared between AA and EA CC patients. Immune cell subsets are aggregated into nine classes with respect to the cytokine network, including CD8 T cells, CD4 T cells (naïve, memory, resting, and activated), B cells (naïve and memory), NK cells (resting and activated), macrophage (M0, M1, M2), dendritic cells (resting, activated), mast cells (resting and activated), neutrophils, and eosinophils; “Aggregate 2” described in the Supplementary Materials of Thorsson et al.^21^ To avoid potential confusion that not all the immune cell subsets are included in this aggregation, we re‐normalized the immune cellular fractions so that they sum to 100%. Immune cellular fraction was then modeled using race, sex, disease stage, and age, which was presented as a dichotomous variable above and below 55 years. Specific lymphocyte and macrophage subtypes were then estimated for AA and EA patients. Lymphocyte and macrophage cellular fractions were also modeled using race, sex, disease stage, and age.

### 2.3 Characterization of immune gene expressions – TCGA

Gene expressions for immuno‐inhibitors and immuno‐stimulators were obtained from the cBioPortal database (http://www.cbioportal.org/) and compared between EA and AA CC patients. Specifically, gene expressions were utilized in the form of z‐scores, the relative expression of an individual gene in a tumor sample compared to the gene's expression distribution in a reference population of samples, where the reference population is defined as all samples that are diploid for that gene.^24^ Major histocompatibility complex (MHC) gene expression level was also compared. In addition, gene set enrichment analysis of differentially expressed (DE) genes was performed on the activation level of the antigen processing and presentation pathway.

### 2.4 Statistical analysis

Kaplan–Meier curves were used to demonstrate overall survival (OS) and progression free survival (PFS).^25^ Survival curves were compared using the log‐rank test. To assess PFS while controlling for additional covariates, we built Cox Proportional Hazards models on age, gender, and cancer stage.^26^ The proportional hazards assumption was assessed for all the covariates by plotting log‐log Kaplan–Meier survival estimates against the log of time. A multivariable linear regression model was created to determine the association of immune cellular fraction components with race, sex, age, and disease stage used as covariates. DE genes were identified by conducting a t‐test between AA and EA for each gene and adjusting for the multiple testing using the Benjamini‐Hochberg procedure. Multivariate logistic regression models of race were also constructed on immune cell proportions. The reference group for these analyses were AA patients. Gene set enrichment analysis was implemented using a hypergeometric test and the antigen processing and presentation pathway obtained from the KEGG (Kyoto Encyclopedia of Genes and Genomes) pathway database.^27^ All analyses were conducted using R software version 3.5.3. Throughout all analyses, statistical significance was as determined by a criterion of *p* < 0.05. The Institutional Review Board of MUSC approved this analysis.

## 3 RESULTS

### 3.1 Clinical CC patient characteristics and survival

**FIGURE 1:**
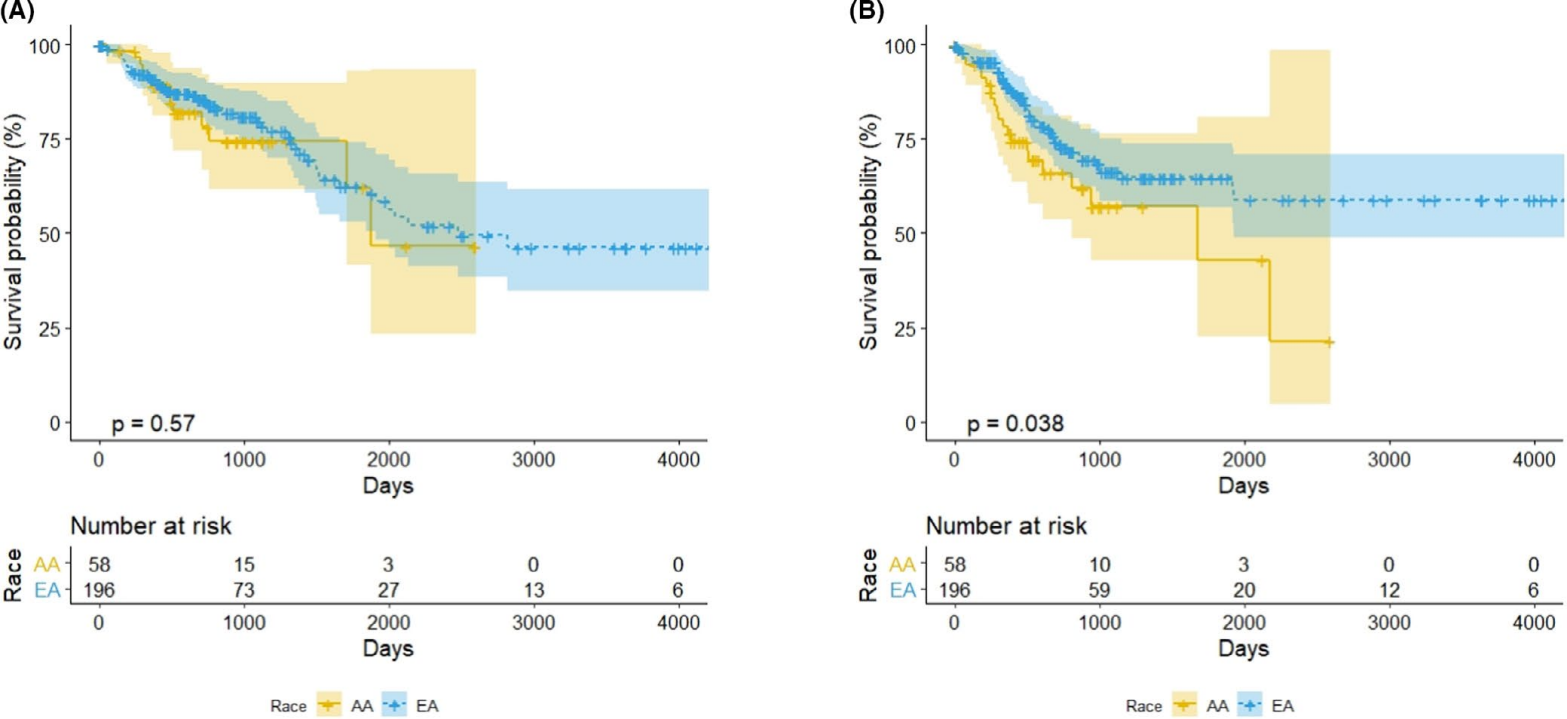
(A) Overall survival and (B) progression‐free survival by race. Yellow and blue colors indicate AA and EA, respectively, and survival curves and 95% confidence intervals are plotted. The *p*‐value in each subfigure indicates the log‐rank test significance

Of the 427 patients with CC from the TCGA data, we analyzed 254 patients meeting inclusion criteria, including 58 AA (23%) and 196 EA (77%). Their demographic characteristics are outlined in Table 1. No differences existed with respect to gender or disease stage. In the CC TCGA cohort, no differences in OS were observed between AA and EA patients (Figure 1A). However, analysis of PFS demonstrated significantly worse outcomes for AA patients compared to EA patients (log‐rank, *p* = 0.038) (Figure 1B). In addition, median progression‐free time for AA was 1678 days while median progression‐free time for EA was not reached. Hazard ratios and 95% confidence interval for race, age, gender, and cancer stage as derived by the multivariable Cox model are shown in Table 2 (A: OS and B: PFS). The proportional hazards assumption was assessed for each covariate in the Cox model (age, gender, and cancer stage) with curves found to be reasonably parallel (Figure S1). For OS, there was no significant difference between EA and AA (*p* = 0.359). AA had worse PFS compared to EA when adjusted for age, gender, and cancer stage (*p* = 0.035). The estimated hazard ratio for AA compared to EA was 1.75 with the 95% confidence interval of (1.04, 2.95). Adjusted OS plots and adjusted PFS plots are provided in Figures S2–S5.

**TABLE 1:** Demographics of African and European American patients

	Overall (*N* = 254)	European American (*N* = 196)	African American (*N* = 58)	*p*‐value
Age				
Mean (SD)	64.9 (13.5)	65.9 (13.2)	61.5 (14.1)	0.04
Median (Min, Max)	67.0 (31.0, 90.0)	68.0 (34.0, 90.0)	61.0 (31.0, 90.0)	
Gender				
Female	126 (49.6%)	95 (48.5%)	31 (53.4%)	0.55
Male	128 (50.4%)	101 (51.5%)	27 (46.6%)	
Stage				
Stage I	42 (16.5%)	34 (17.3%)	8 (13.8%)	0.60
Stage II	94 (37.0%)	75 (38.3%)	19 (32.8%)	
Stage III	81 (31.9%)	61 (31.1%)	20 (34.5%)	
Stage IV	37 (14.6%)	26 (13.3%)	11 (19.0%)	

**TABLE 2:** Cox regression analysis result: Hazard ratios and 95% confidence intervals of race, age, gender and cancer stage for (A) overall survival and (B) progression‐free survival

	Hazard ratio	95% CI	*p*
(A) Overall survival			
Race			
EA	1.00		
AA	1.34	(0.71, 2.54)	0.359
Age (years)			
<65	1.00		
≥65	1.55	(0.91, 2.66)	0.110
Gender			
Female	1.00		
Male	1.48	(0.88, 2.47)	0.138
Cancer stage			
Local (Stage 1)	1.00		
Advanced (Stage 2–4)	2.29	(0.83, 6.32)	0.111

	Hazard ratio	95% CI	*p*
(B) Progression‐free survival			
Race			
EA	1.00		
AA	1.75	(1.04, 2.95)	0.035*
Age (years)			
<65	1.00		
≥65	0.69	(0.43, 1.11)	0.130
Gender			
Female	1.00		
Male	1.82	(1.12, 2.95)	0.015**
Cancer stage			
Local (Stage 1)	1.00		
Advanced (Stage 2–4)	3.60	(1.31, 9.88)	0.013*

* *p* < 0.05;

** *p* < 0.01;

*** *p* < 0.001.

### 3.2 Racial differences in the CRC immune microenvironment

**FIGURE 2:**
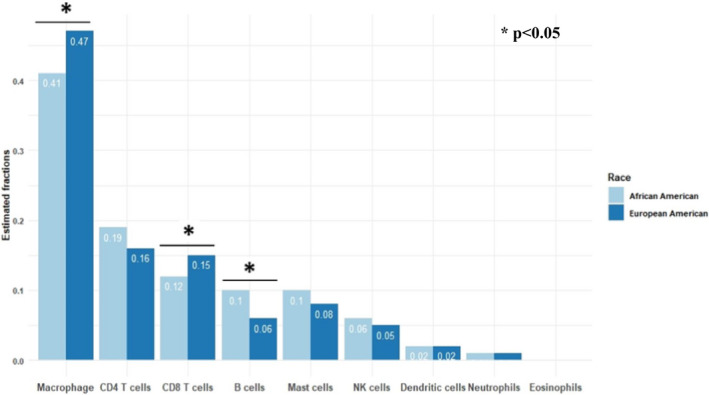
Immune cellular fraction estimates by race

Using the analytic strategy described by Thorsson,^21^ immune populations were identified in tumors from both AA and EA CC patients. This analysis identified significant differences in gene expression associated with specific immune cell populations. Compared with tumors from EA patients, tumors from AA patients showed a greater proportion of B cells (*p* < 0.01) and a decreased proportion of macrophages (*p* < 0.01) and CD8 T cells (*p* = 0.03) as compared to EA patients (Figure 2 and Table S1). As differences were identified for CD8 T cells, B cells, and macrophages, subsets of these cell types were further analyzed for association with race (Table 3). AA patient tumors have more memory B (*p* < 0.01) and plasma cells (*p* < 0.01) compared with EA patient tumors. In addition to fewer macrophages, pro‐inflammatory macrophages with an M1 phenotype were also reduced in tumors from AA patients (*p* < 0.01). Although no differences were observed in the overall NK cell populations, the prevalence of resting NK cells was greater in the AA tumors (*p* < 0.01) while the activated NK cell population was greater in EA tumors (*p* < 0.01). Significant differences in gene expression persisted when controlling for sex, age, and disease stage (Tables S2 and S3). A multivariate logistic regression model of race on the immune cell proportions (Table S4) further confirms an association with increased B cell populations in AA patients (*p* = 0.019). Taken together, the immunogenomics analysis suggested that AA patients have greater pro‐tumorigenic immune characteristics compared to EA patients. Moreover, this analysis suggested that macrophages in EA tumors had a greater proportion with a M1 phenotype that is typically associated with a favorable immune response.

**TABLE 3:** Lymphocyte and macrophage subset fraction by race. In the last column, *, **, and *** indicate 0.01 < *p*‐value ≤0.05, 0.001 < *p*‐value ≤0.01, and *p*‐value ≤0.001, respectively

	All (*N* = 254)	European American (*N* = 196)	African American (*N* = 58)	*p*‐value
B cells‐naive	0.12	0.12	0.10	0.10
B cells‐memory	0.24	0.23	0.25	<0.001^***^
Plasma cells	0.00	0.00	0.00	<0.001^***^
T cells‐CD8	0.06	0.05	0.08	0.03^*^
T cells‐CD4 naive	0.04	0.04	0.05	0.15
T cells‐CD4 memory resting	0.04	0.03	0.06	0.14
T cells‐CD4 memory activated	0.41	0.42	0.36	0.18
T cells‐follicular helper	0.01	0.01	0.01	0.20
T cells‐regulatory	0.08	0.07	0.08	0.65
T cells‐gamma delta	0.01	0.01	0.01	0.59
NK cells‐resting	0.00	0.00	0.00	<0.001^***^
NK cells‐activated	0.12	0.12	0.10	<0.01^**^
Monocytes	0.24	0.23	0.25	0.26
Macrophages‐M0	0.00	0.00	0.00	0.13
Macrophages‐M1	0.06	0.05	0.08	<0.001^***^
Macrophages‐M2	0.04	0.04	0.05	0.49

### 3.3 Racial differences in CRC immune regulatory pathways

**FIGURE 3:**
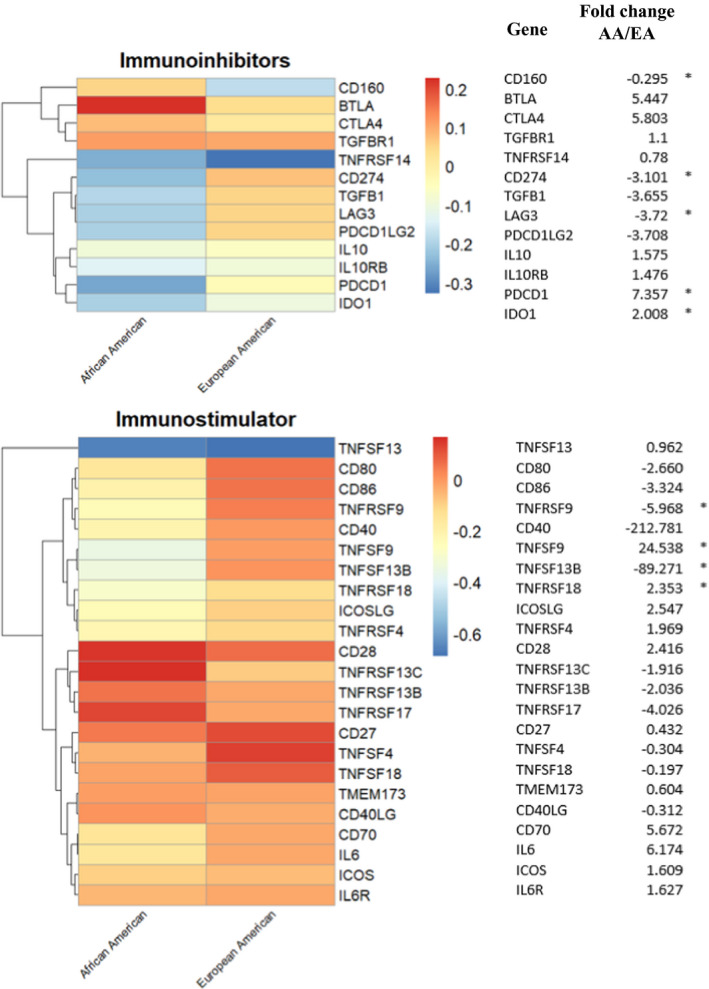
Association of immune modulating gene expressions with race. The heatmap presents averaged gene expressions (*z*‐scores) for immuno‐inhibitors and immuno‐stimulators in each racial group. Log2 fold changes are provided, where * denotes *t*‐test *p*‐value <0.05, indicating the significant racial differences in expressions between the two racial group

Considering the observed racial differences in the CC tumor infiltrating immune cell populations, we also wanted to explore possible alterations in tumor immune regulatory pathways. Thus, we investigated the expression of previously reported immunostimulatory and immunoinhibitory gene profiles in both EA and AA patients (Figure 3). In general, AA patients demonstrated a significantly decreased expression of immunoinhibitory genes compared with EA patients including IDO1 (*p* < 0.01), PD‐1 (PDCD1) (*p* = 0.01), PD‐L1 (CD274) (*p* < 0.01), and LAG3 (*p* = 0.01). Conversely, AA CC tumors expressed relatively high levels of the immune regulatory molecule: CD160 (*p* = 0.02). In comparison, both racial groups expressed relatively high levels of other immune regulatory molecules such as CD28 and CD27. CC tumors from AA patients manifested decreased expression of CD40, 4‐1BB ligand (TNFSF9), 4‐1BB (TNFRSF9), glucocorticoid‐induced tumor necrosis factor receptor (TNFRSF18; GITR) and increased expression of B‐cell activating factor (TNFSF13B) when compared to EA patients. Taken together, this immunogenomic analysis suggests higher expression of immune regulatory genes in AA versus EA CC patients. Functional descriptions of differentially expressed genes are noted in Table S5.

### 3.4 Racial differences in CRC MHC I and II expression

**FIGURE 4:**
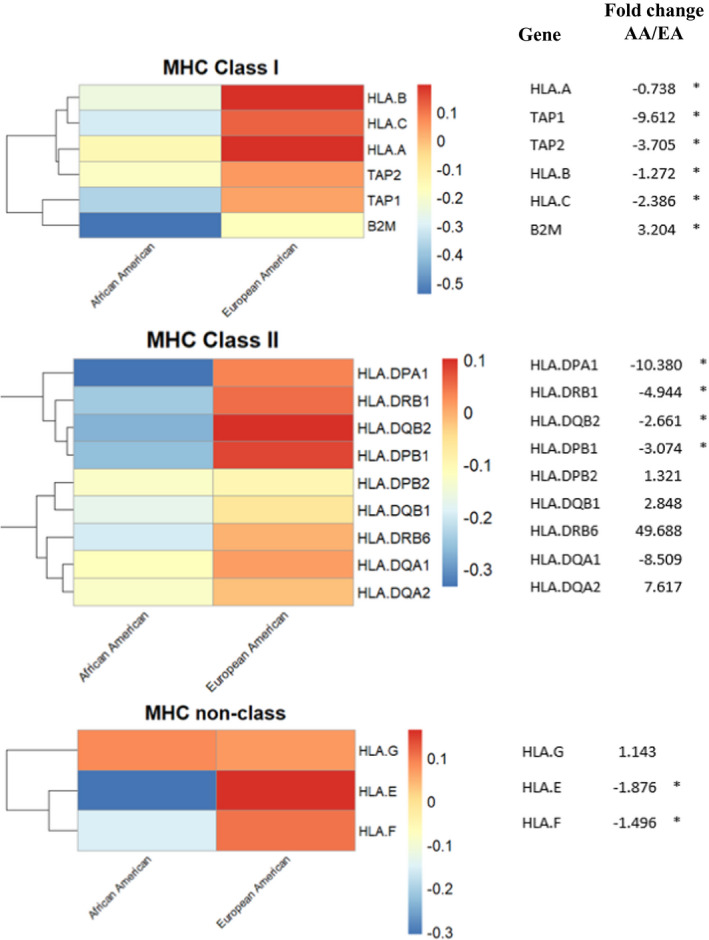
Association of MHC gene expression with race. The heatmap presents averaged gene expressions (z‐scores) for major histocompatibility complex (MHC) genes in each racial group. Log2 fold changes are provided, where * denotes *p*‐value <0.05, indicating the significant racial differences in expressions between the two racial groups

To further define potential drivers of the differences in immune regulation in AA versus EA CC tumors, we investigated the relative expression of key mediators of antigen presentation including MHC class I, and II components (Figure 4 and Figure S1). MHC class I molecules including HLA A, B, and C were decreased in AA CC tumors and a similar pattern was observed for MHC class II molecules. Of the components for antigen presentation to the MHC class I pathway, TAP1 demonstrated the greatest reduction in expression (−9.6‐fold) relative to EA CRCs. A correlation between MHC molecules and immune cell markers is presented in Figure S6. In addition, low expression of MHC molecules such as for TAP1 correlated with worse patient survival further supporting the clinical relevance of the racial differences in MHC expression (Figures S7 and S8). In addition to the analysis of the individual gene expression of MHC I and MHC II components, we also performed an enrichment analysis of the genes differentially expressed between AA and EA patients, with respect to the antigen processing and presentation pathway from the KEGG pathway database. We found that there is statistically significant enrichment of the DE genes for the antigen processing and presentation pathway (*p*‐value =0.009).

## 4 DISCUSSION

Investigating patient and tumor features that underlie well‐established CRC racial disparities holds promise to uncover potential deficiencies in the tumor immune response of AA versus EA patients. Defining underlying cellular and molecular racial factors that promote poorer outcomes in AA patients may enable the design of therapeutic interventions to overcome disparities. Emerging studies suggest alterations in T cell presence and function in AAs may contribute to CRC disparities.^4^, ^28^ Our group previously reported that low immune infiltrate in tumors from AA patients was more predictive of poor outcome.^4^ To extend this line of investigation, we applied an immunogenomic analysis strategy to investigate CC racial differences in gene expression data from TCGA.

Our evaluation of the tumor immune microenvironment revealed striking differences between AA and EA CC. AA CC tumors displayed a decreased fraction of macrophages and CD8 T cells and an increased fraction of B cells compared with EA tumors in the tumor microenvironment, possibly suggesting a more favorable immune environment for CD8‐mediated antitumor immunity in EA cancers. The cellular outcomes persisted when controlling for sex, age, and disease stage. (Tables S2 and S3). Multivariate logistic regression fit on race also demonstrated an increased B‐cell fraction (Table S4). Further differences were identified with respect to the expression of immunostimulatory and immunoinhibitory genes as well as MHC class I and class II molecules. For example, gene expression analysis demonstrated increased expression of immune regulatory molecules in EA patients including genes encoding for PD‐L1 and PD‐1. While expression of these inhibitory molecules is often thought of as a poor prognostic factor, molecules such as PD‐L1 may be preferentially absent on tumors without immune infiltration.^29^ Thus, presence of immune inhibitory molecules may reflect the presence of immune cells and a state of immune equilibrium. Interestingly, a prior report found that African ancestry has also been associated with decreased PD‐L1 expression across cancer types including colorectal adenocarcinoma, breast cancer, head, and neck squamous cell cancer and papillary thyroid cancer.^21^ In addition, the elevated macrophages, particularly M1 macrophages, is suggestive of a more favorable immune environment in EA CRCs.

The reduced expression of MHC class I and II in AA patient tumors was similarly noteworthy. While loss of MHC class I and II expression may reflect alterations in antigen processing in tumor or professional antigen presenting cells, it could also simply reflect a general reduced immune infiltration into the tumor, especially in the possible reduction of IFNγ. Imaging studies defining MHC class I and II expression on different immune cells in the tumor will be necessary to address these possibilities. Given the critical role of tumoral antigen presentation in sustaining immune‐based therapies,^30^, ^31^, ^32^ this finding is likely to be a key area of investigation for CRC racial disparities.

Seminal studies have established a strong relationship between increased peritumoral lymphocyte density and survival in CRC that exceeded the prognostic ability of TNM stage.^11^ Wallace and colleagues from our institution supported the association between lymphocyte density and survival yet also identified a subset of young AA patients who fared poorly despite a high lymphocyte density suggesting that lymphocyte function and/or gene expression rather than density of cells may play a role in survival.^6^ In addition, previous work by Basa and colleagues demonstrated a significant decrease in Granzyme B+ infiltration for AA CRCs suggesting a decreased cytotoxic effect.^17^ Our current analysis builds on these observations suggesting that the antigen presentation process and T cell activity are globally reduced in AA patients resulting in less active cytotoxic T cells. The decreased expression of checkpoint mediators in AA tumors suggests that AA tumors compared with EA tumors have not activated specific T cell inhibitory pathways.

The results of this study must be evaluated in the context of its data source and study design. While TCGA is among the largest repositories of molecular cancer data available, the sample size specific to CC is relatively small with the majority of tumor samples collected from EA patients which increases the possibility of type II error within our analyses. For example, the lack of survival differences between early and late stage of disease in our analysis is likely attributed to a type 2 error. Though a wealth of molecular information is available through TCGA, certain information such as microsatellite stability are not readily available. Additionally, granular, patient‐level socioeconomic and clinical data regarding patient treatment is unavailable in TCGA which allows for the possibility that differences in survival could be attributable to differences in access to care and treatment received. With respect to racial differences in PFS and tumor immune response, the mechanism by which racial differences in tumor immune response may underlie disparate clinical outcomes remains to be elucidated. Future study of a larger study population may allow for characterization of the underlying mechanism for these differences via a race stratified investigation of the relationships between specific immunogenomic signatures and survival outcomes.

## 5 CONCLUSIONS

In summary, this secondary analysis of a large, publicly available CC molecular data repository has identified significant differences in the tumor immune microenvironment of AA and EA tumor samples in the setting of a decreased PFS for AA patients. While exploratory in nature, these differences call attention to varied immunological signatures for AA CC as compared to EA CC that may play a role in explaining not only the clinical differences in outcome between the groups, but also provide possible targeted therapeutic opportunities.

## 6 Availability of data and materials

All data are publicly available as noted in the methods section.

## 7 Ethics approval and consent to participate

As these data are part of a de‐identified, publicly available database, this does not constitute human subjects research and informed consent was not applicable. The Institutional Review Board of the Medical University of South Carolina approved this analysis.

## Conflict of interests

No conflicts to disclose.

## Supporting information

Supplementary Material
