# Supplementary material for: Differential immune signatures in the tumor microenvironment are associated with colon cancer racial disparities
Source: Cancer Med. 2021 Feb 9;10(5):1805–14. doi: 10.1002/cam4.3753 (PMC7940243; doi:10.1002/cam4.3753)
Supplement: Supplementary file 1 — Supplementary Material [file CAM4-10-1805-s001.zip › cam43753-sup-0001-FigS1.pptx]

## Slide 1
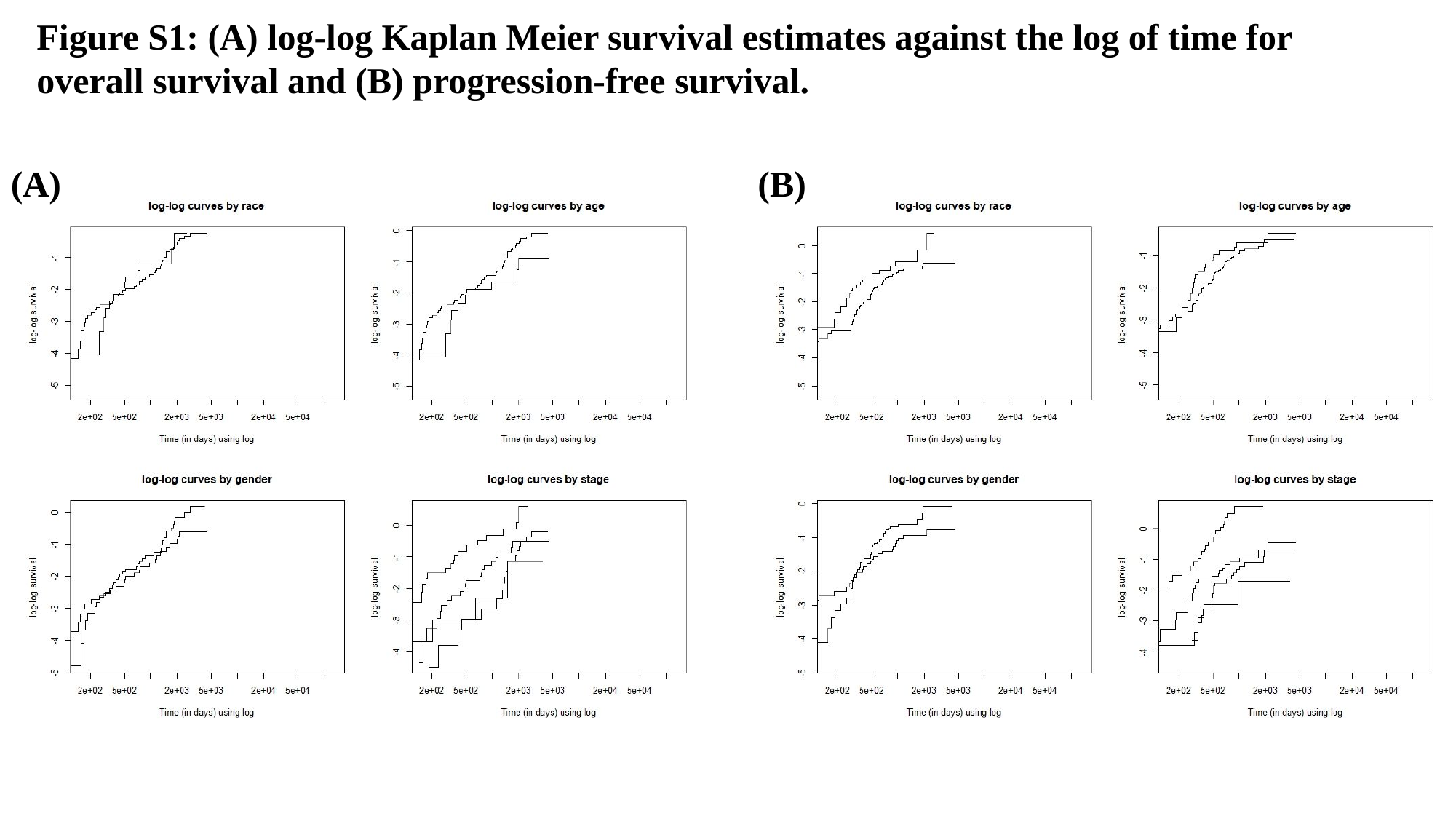

Figure S1: (A) log-log Kaplan Meier survival estimates against the log of time for overall survival and (B) progression-free survival.
(B)
(A)
