# Supplementary material for: Differential immune signatures in the tumor microenvironment are associated with colon cancer racial disparities
Source: Cancer Med. 2021 Feb 9;10(5):1805–14. doi: 10.1002/cam4.3753 (PMC7940243; doi:10.1002/cam4.3753)
Supplement: Supplementary file 1 — Supplementary Material [file CAM4-10-1805-s001.zip › cam43753-sup-0003-FigS3.pptx]

## Slide 1
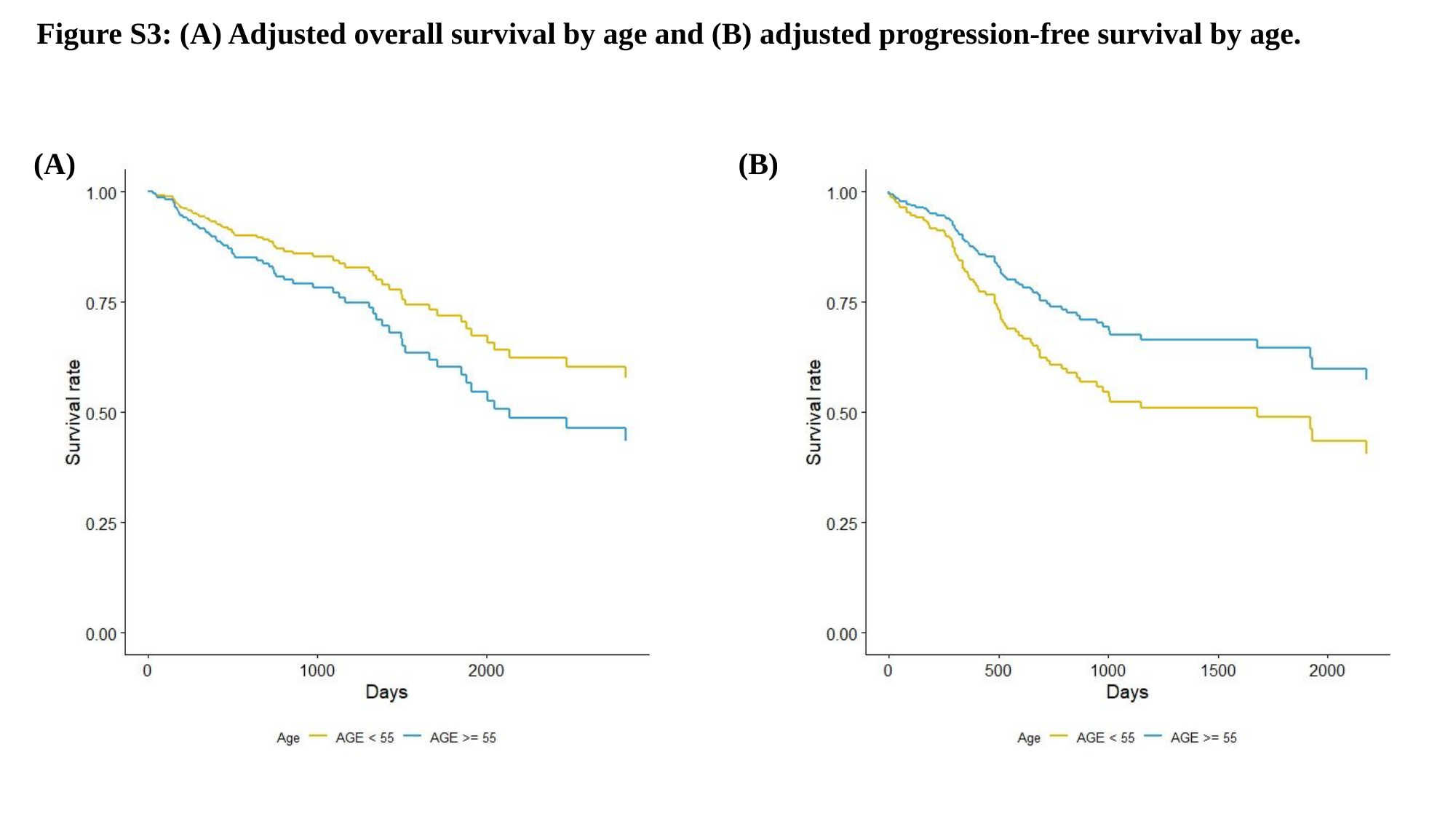

Figure S3: (A) Adjusted overall survival by age and (B) adjusted progression-free survival by age.
(A)
(B)
