# Supplementary material for: Differential immune signatures in the tumor microenvironment are associated with colon cancer racial disparities
Source: Cancer Med. 2021 Feb 9;10(5):1805–14. doi: 10.1002/cam4.3753 (PMC7940243; doi:10.1002/cam4.3753)
Supplement: Supplementary file 1 — Supplementary Material [file CAM4-10-1805-s001.zip › sm_0001-TableS1-S5..docx]

**Supplemental Table 1: Lymphocyte and macrophage fraction by race.** In the last column, *, **, and *** indicate 0.01 < p-value ≤ 0.05, 0.001 < p-value ≤ 0.01, and p-value ≤ 0.001, respectively.

|  | **All**  **(N=254)** | **European American**  **(N=196)** | **African American**  **(N=58)** | **p-value** |
| --- | --- | --- | --- | --- |
| **T cells CD8** | 0.14 | 0.15 | 0.12 | * 0.03 |
| **T cells CD4** | 0.17 | 0.16 | 0.19 | 0.20 |
| **B cells** | 0.07 | 0.06 | 0.10 | ** 0.002 |
| **NK cells** | 0.05 | 0.05 | 0.06 | 0.28 |
| **Macrophage** | 0.45 | 0.47 | 0.41 | ** 0.003 |
| **Dendritic cells** | 0.02 | 0.02 | 0.02 | 0.67 |
| **Mast cells** | 0.09 | 0.08 | 0.10 | 0.36 |
| **Neutrophils** | 0.01 | 0.01 | 0.01 | 0.40 |
| **Eosinophils** | 0.00 | 0.00 | 0.00 | 0.69 |

**Supplemental Table 2: Lymphocyte and macrophage subset fraction by race, adjusted for age, gender and cancer stage.** In the last column, *, **, and *** indicate 0.01 < p-value ≤ 0.05, 0.001 < p-value ≤ 0.01, and p-value ≤ 0.001, respectively.

|  | **All**  **(N=254)** | **European American**  **(N=196)** | **African American**  **(N=58)** | **p-value** |
| --- | --- | --- | --- | --- |
| **B cells-naive** | 0.12 | 0.12 | 0.10 | 0.097 |
| **B cells-memory** | 0.24 | 0.23 | 0.25 | *** <0.001 |
| **Plasma cells** | 0.00 | 0.00 | 0.00 | *** <0.001 |
| **T cells-CD8** | 0.06 | 0.05 | 0.08 | * 0.033 |
| **T cells-CD4 naive** | 0.04 | 0.04 | 0.05 | 0.154 |
| **T cells-CD4 memory resting** | 0.04 | 0.03 | 0.06 | 0.143 |
| **T cells-CD4 memory activated** | 0.41 | 0.42 | 0.36 | 0.180 |
| **T cells-follicular helper** | 0.01 | 0.01 | 0.01 | 0.200 |
| **T cells-regulatory** | 0.08 | 0.07 | 0.08 | 0.647 |
| **T cells-gamma delta** | 0.01 | 0.01 | 0.01 | 0.587 |
| **NK cells-resting** | 0.00 | 0.00 | 0.00 | *** <0.001 |
| **NK cells-activated** | 0.12 | 0.12 | 0.10 | ** 0.002 |
| **Monocytes** | 0.24 | 0.23 | 0.25 | 0.256 |
| **Macrophages-M0** | 0.00 | 0.00 | 0.00 | 0.130 |
| **Macrophages-M1** | 0.06 | 0.05 | 0.08 | *** <0.001 |
| **Macrophages-M2** | 0.04 | 0.04 | 0.05 | 0.486 |

**Supplemental Table 3: Lymphocyte and macrophage fraction by race, adjusted for age, gender and cancer stage.** In the last column, *, **, and *** indicate 0.01 < p-value ≤ 0.05, 0.001 < p-value ≤ 0.01, and p-value ≤ 0.001, respectively.

|  | **All**  **(N=254)** | **European American**  **(N=196)** | **African American**  **(N=58)** | **p-value** |
| --- | --- | --- | --- | --- |
| **T cells CD8** | 0.14 | 0.15 | 0.12 | * 0.050 |
| **T cells CD4** | 0.17 | 0.16 | 0.19 | 0.180 |
| **B cells** | 0.07 | 0.06 | 0.10 | ** 0.004 |
| **NK cells** | 0.05 | 0.05 | 0.06 | 0.215 |
| **Macrophage** | 0.45 | 0.47 | 0.41 | ** 0.003 |
| **Dendritic cells** | 0.02 | 0.02 | 0.02 | 0.618 |
| **Mast cells** | 0.09 | 0.08 | 0.10 | 0.352 |
| **Neutrophils** | 0.01 | 0.01 | 0.01 | 0.339 |
| **Eosinophils** | 0.00 | 0.00 | 0.00 | 0.594 |

**Supplemental Table 4: Multiple logistic regression model fit of race on immune cell proportions.** In the last column, *, **, and *** indicate 0.01 < p-value ≤ 0.05, 0.001 < p-value ≤ 0.01, and p-value ≤ 0.001, respectively.

|  | Estimate | Std. Error | z value | p-value |
| --- | --- | --- | --- | --- |
| (Intercept) | 2.018 | 1.822 | 1.107 | 0.268 |
| CD8 T cells | 0.005 | 0.003 | 1.45 | 0.147 |
| CD4 T cells | -0.003 | 0.003 | -0.893 | 0.372 |
| B cells | -0.007 | 0.003 | -2.353 | *0.019 |
| NK cells | -0.002 | 0.002 | -0.967 | 0.334 |
| Macrophage | 0.003 | 0.004 | 0.763 | 0.446 |
| Dendritic cells | 0.001 | 0.003 | 0.325 | 0.745 |
| Mast cells | -0.004 | 0.003 | -1.299 | 0.194 |
| Neutrophils | 0.006 | 0.007 | 0.881 | 0.378 |
| Eosinophils | 0.003 | 0.012 | 0.277 | 0.782 |

**Supplemental Table 5: Functional significance of distinct genes in AA vs. EA CRC**

| **Gene** | **Functional significance** | **Clinical evidence** |
| --- | --- | --- |
| **Downregulated** |  |  |
| **IDO1** | Impacts T-cell behavior via activity in tryptophan metabolism | Targeted therapies studied in melanoma and glioblastoma(33) |
| **PDCD1 (aka PD1)** | Expressed on pro-B-cells and plays role in cell differentiation | PD-1/PD-L1 pathway potential therapeutic target for CRC(34) |
| **CD274 (aka PD-L1)** | T cell inhibitory receptor ligand | PD-1/PD-L1 pathway potential therapeutic target for CRC(34) |
| **LAG3** | Role in lymphocyte activation | Blockade studied in treatment of CRC liver metastases(35) |
| **TNFSF9 (aka CD137L)** | Cytokine associated with optimizing CD8 T-cell response | Differential levels in colon vs. rectal cancer with possible implications for carcinogenesis(36) |
| **TNFRSF9 (aka CD137)** | Associated with clonal expansion, survival and development of T cells. | Differential levels in colon vs. rectal cancer with possible implications for carcinogenesis(36) |
| **TNFRSF18 (aka GITR)** | Plays role in self-tolerance mediated by CD25(+)CD4(+) regulatory T cells | Potential role in inhibiting Treg function concurrent with CD8 cell activation(37) |
| **Upregulated** |  |  |
| **CD160 (aka NK1)** | Binds MHC Class I. Expressed on intestinal lymphocytes associated with HVEM. | Associated with improved clinical outcome in hepatocellular carcinoma(38) |
| **TNFSF13B** | Cytokine in TNF ligand family with role in proliferation and differentiation of B cells. | Associated with poor outcome in study of topoisomerase inhibitors in CRC(39) |

References:

Liu M, Wang X, Wang L, et al. Targeting the IDO1 pathway in cancer: from bench to bedside. J Hematol Oncol. 2018;11(1):100.

Yaghoubi N, Soltani A, Ghazvini K, Hassanian SM, Hashemy SI. PD‐1/ PD‐L1 blockade as a novel treatment for colorectal cancer. Biomed Pharmacother. 2019;110:312–318.

Zhou G, Noordam L, Sprengers D, et al. Blockade of LAG3 enhances responses of tumor‐infiltrating T cells in mismatch repair‐proficient liver metastases of colorectal cancer. Oncoimmunology. 2018;7(7):e1448332.

Dimberg J, Hugander A, Wagsater D. Expression of CD137 and CD137 ligand in colorectal cancer patients. Oncol Rep. 2006;15(5):1197–1200.

Knee DA, Hewes B, Brogdon JL. Rationale for anti‐GITR cancer immunotherapy. Eur J Cancer. 2016;67:1–10.

Sun H, Xu J, Huang Q, et al. Reduced CD160 expression contributes to impaired NK‐cell function and poor clinical outcomes in patients with HCC. Cancer Res. 2018;78(23):6581–6593.

Bedi D, Henderson HJ, Manne U, Samuel T. Camptothecin induces PD‐L1 and immunomodulatory cytokines in colon cancer cells. Medicines (Basel). 2019;6(2):51.
