# Supplementary figures and images for: Differential immune signatures in the tumor microenvironment are associated with colon cancer racial disparities
Source: Cancer Med. 2021 Feb 9;10(5):1805–14. doi: 10.1002/cam4.3753 (PMC7940243; doi:10.1002/cam4.3753)

## Slide 1
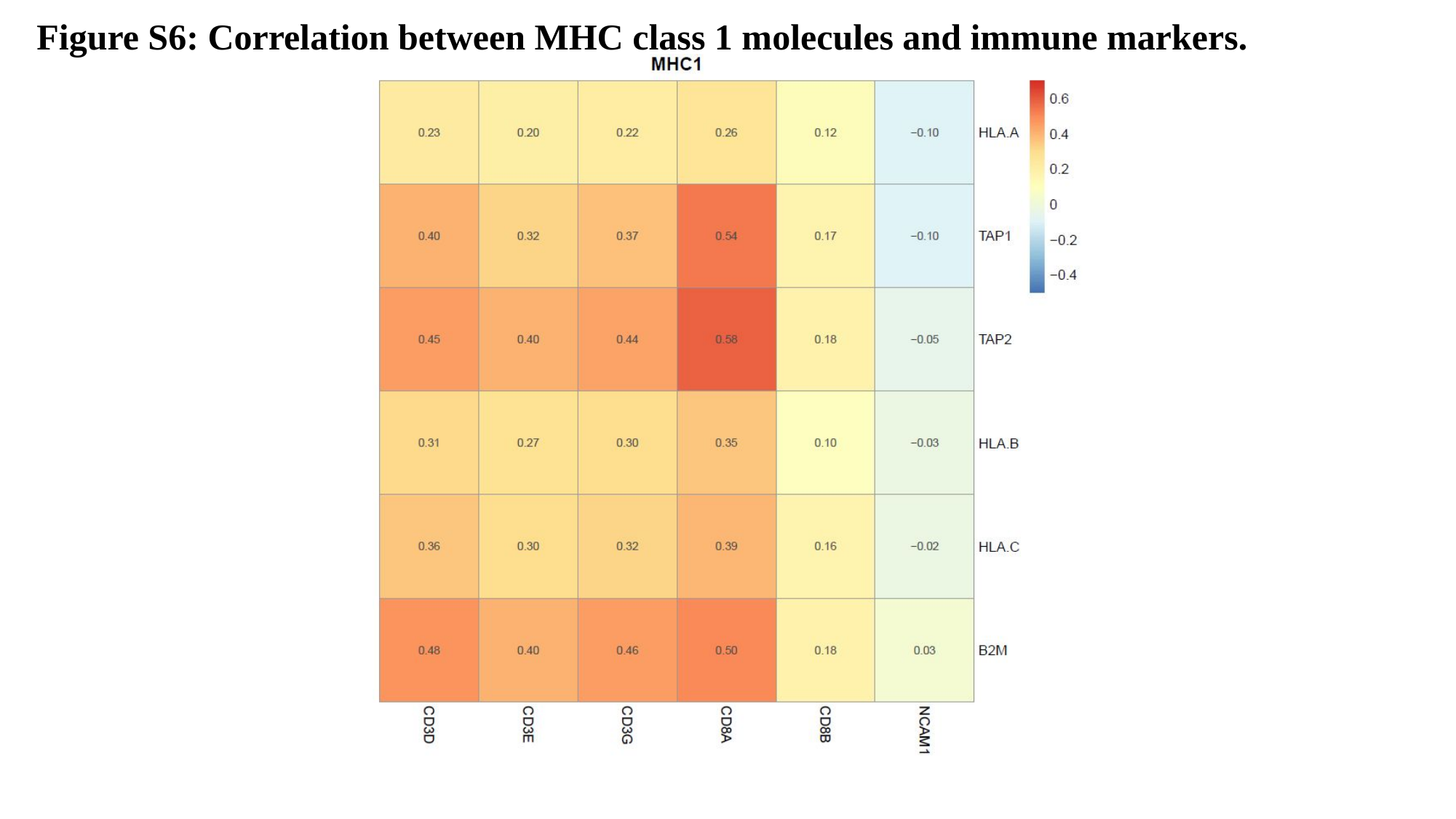

Figure S6: Correlation between MHC class 1 molecules and immune markers.

Supplement: Supplementary file 1 — Supplementary Material [file CAM4-10-1805-s001.zip › cam43753-sup-0006-FigS6.pptx]

## Slide 1
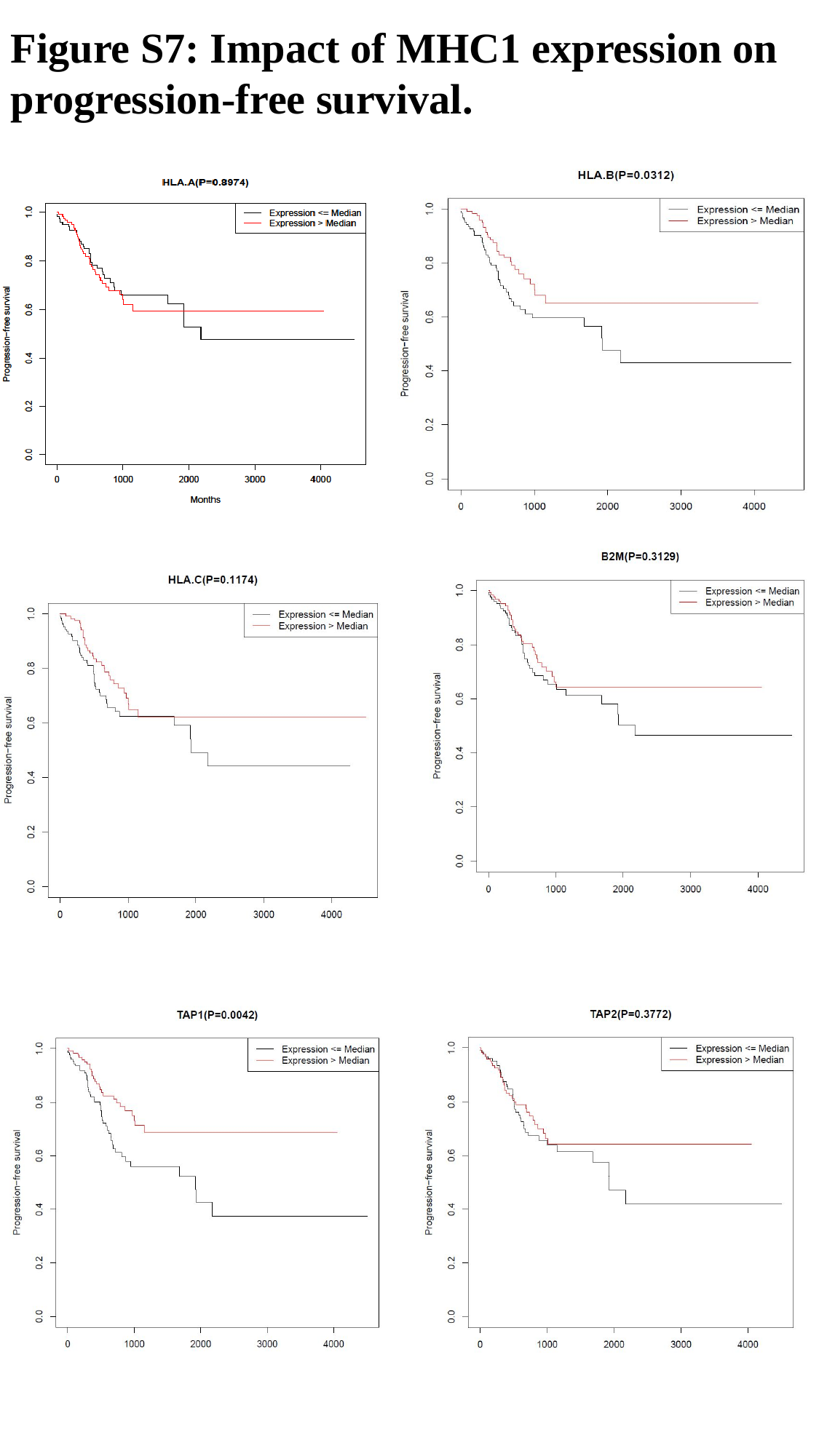

Figure S7: Impact of MHC1 expression on progression-free survival.

Supplement: Supplementary file 1 — Supplementary Material [file CAM4-10-1805-s001.zip › cam43753-sup-0007-FigS7.pptx]

## Slide 1
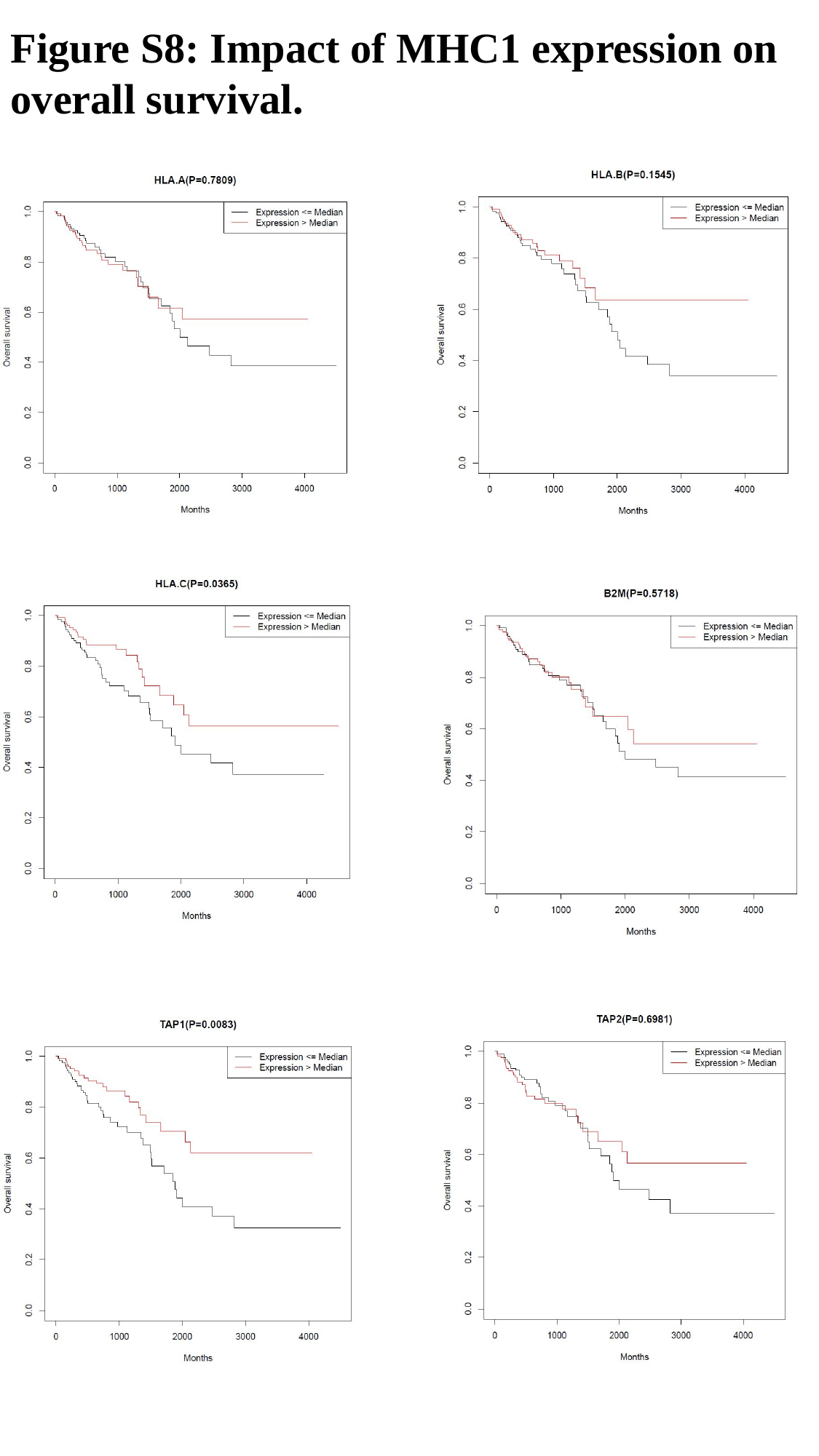

Figure S8: Impact of MHC1 expression on overall survival.

Supplement: Supplementary file 1 — Supplementary Material [file CAM4-10-1805-s001.zip › cam43753-sup-0008-FigS8.pptx]
